# A Simple Cervicovaginal Epigenetic Test for Screening and Rapid Triage of Women With Suspected Endometrial Cancer: Validation in Several Cohort and Case/Control Sets

**DOI:** 10.1200/JCO.22.00266

**Published:** 2022-08-24

**Authors:** Chiara Herzog, Fátima Marín, Allison Jones, Iona Evans, Daniel Reisel, Elisa Redl, Lena Schreiberhuber, Sonia Paytubi, Beatriz Pelegrina, Álvaro Carmona, Paula Peremiquel-Trillas, Jon Frias-Gomez, Marta Pineda, Joan Brunet, Jordi Ponce, Xavier Matias-Guiu, Silvia de Sanjosé, Laia Alemany, Adeola Olaitan, Michael Wong, Davor Jurkovic, Emma J. Crosbie, Adam N. Rosenthal, Line Bjørge, Michal Zikan, Lukas Dostalek, David Cibula, Karin Sundström, Joakim Dillner, Laura Costas, Martin Widschwendter

**Affiliations:** ^1^ European Translational Oncology Prevention and Screening (EUTOPS) Institute, Universität Innsbruck, Innsbruck, Austria; ^2^ Institute for Biomedical Aging Research, Universität Innsbruck, Innsbruck, Austria; ^3^ Hereditary Cancer Group, Catalan Institute of Oncology, IDIBELL, ONCOBELL Program, L'Hospitalet, Barcelona, Spain; ^4^ Consortium for Biomedical Research in Cancer—CIBERONC, Carlos III Institute of Health, Madrid, Spain; ^5^ Department of Women's Cancer, University College London, London, United Kingdom; ^6^ Cancer Epidemiology Research Programme, Catalan Institute of Oncology, IDIBELL, L'Hospitalet de Llobregat, Barcelona, Spain; ^7^ Hereditary Cancer Group, Catalan Institute of Oncology, IDIBGI, Girona, Spain; ^8^ Department of Gynecology, Hospital Universitari de Bellvitge, IDIBELL, Hospitalet de Llobregat, Barcelona, Spain; ^9^ Department of Pathology, Hospital Universitari de Bellvitge, IDIBELL, Hospitalet de Llobregat, Barcelona, Spain; ^10^ ISGlobal, Barcelona, Spain; ^11^ Consortium for Biomedical Research in Epidemiology and Public Health—CIBERESP, Carlos III Institute of Health, Madrid, Spain; ^12^ University College Hospital, London, United Kingdom; ^13^ Department of Obstetrics and Gynaecology, Manchester Academic Health Science Center, St Mary's Hospital, Manchester University NHS Foundation Trust, Manchester, United Kingdom; ^14^ Gynaecological Oncology Research Group, Division of Cancer Sciences, University of Manchester, Manchester, United Kingdom; ^15^ Department of Obstetrics and Gynaecology, Haukeland University Hospital, Bergen, Norway; ^16^ Department of Clinical Science, Center for Cancer Biomarkers CCBIO, University of Bergen, Bergen, Norway; ^17^ Department of Gynecology and Obstetrics, Charles University in Prague, First Faculty of Medicine and Bulovka University Hospital, Czech Republic; ^18^ Department of Obstetrics and Gynecology, First Faculty of Medicine, Gynaecologic Oncology Center, Charles University in Prague, General University Hospital in Prague, Prague, Czech Republic; ^19^ Division of Pathology, Department of Laboratory Medicine, Karolinska Institutet, Stockholm, Sweden; ^20^ Department of Women's and Children's Health, Karolinska Institutet, Stockholm, Sweden

## Abstract

Endometrial cancer (EC) incidence has been rising over the past 10 years. Delays in diagnosis reduce survival and necessitate more aggressive treatment. We aimed to develop and validate a simple, noninvasive, and reliable triage test for EC to reduce the number of invasive diagnostic procedures and improve patient survival.

**METHODS:**

We developed a test to screen and triage women with suspected EC using 726 cervical smear samples from women with and without EC, and validated the test in 562 cervicovaginal samples using three different collection methods (cervical smear: n = 248; vaginal swab: n = 63; and self-collection: n = 251) and four different settings (case/control: n = 388; cohort of women presenting with postmenopausal bleeding: n = 63; a cohort of high-risk women with Lynch syndrome: n = 25; and a nested case/control setting from a screening cohort and samples taken up to 3 years before EC diagnosis: n = 86).

**RESULTS:**

We describe the Women's cancer risk IDentification – quantitative polymerase chain reaction test for Endometrial Cancer (WID-qEC), a three-marker test that evaluates DNA methylation in gene regions of *GYPC* and *ZSCAN12*. In cervical, self-collected, and vaginal swab samples derived from symptomatic patients, it detected EC with sensitivities of 97.2% (95% CI, 90.2 to 99.7), 90.1% (83.6 to 94.6), and 100% (63.1 to 100), respectively, and specificities of 75.8% (63.6 to 85.5), 86.7% (79.3 to 92.2), and 89.1% (77.8 to 95.9), respectively. The WID-qEC identified 90.9% (95% CI, 70.8 to 98.9) of EC cases in samples predating diagnosis up to 1 year. Test performance was similar across menopausal status, age, stage, grade, ethnicity, and histology.

**CONCLUSION:**

The WID-qEC is a noninvasive reliable test for triage of women with symptoms suggestive of ECs. Because of the potential for self-collection, it could improve early diagnosis and reduce the reliance for in-person visits.

## INTRODUCTION

Endometrial cancer (EC) is among the tumor types with the sharpest rising incidence over the past 10 years.^1,2^ Abnormal bleeding, defined as any postmenopausal, intermenstrual, or persistent heavy menstrual bleeding, is the lead symptom. EC patient survival is strongly dependent on stage at diagnosis,^3^ with delays in diagnosis and treatment resulting in significant adverse impacts on survival.^4^ The current route of diagnosis for suspected EC is transvaginal ultrasound (TVUS) followed by hysteroscopy and endometrial biopsy.^5^ During the COVID-19 lockdown, referrals via the 2-week-wait urgent pathway for suspected cancer in England, United Kingdom, decreased by up to 84%. A 3-month delay in EC diagnosis in England alone has been suggested to cause a loss of 6,305 life-years.^6^ A rapid triage modality that could rule out malignancies without the need for initial specialist referral could improve patient care and reduce time to diagnosis.

**CONTEXT**

**Key Objective**
Endometrial cancer (EC) is among the most common cancers in women. Tests for suspected EC are invasive, expensive, and require a specialist setting. Assessing DNA methylation in 1,288 cervicovaginal specimens, we developed and validated the Women's cancer risk IDentification – quantitative polymerase chain reaction test for Endometrial Cancer (WID-qEC), a patient-friendly tool for screening and triaging women with suspected EC.
**Knowledge Generated**
The WID-qEC identified 100% of ECs within a cohort of women presenting with postmenopausal bleeding at 89% specificity. Moreover, the test could also be performed on self-collected samples and resulted in higher accuracy than ultrasound and somatic mutation analysis in our setting.
**Relevance**
The WID-qEC may enable rapid, noninvasive screening and triage for symptomatic women at greatest risk, avoiding invasive investigations in healthy women. Future prospective studies will confirm whether it could be used to replace ultrasound to triage women with suspected EC, and further clarify its utility for screening in general and high-risk populations.

Current triage investigations available for suspected EC suffer from several limitations. Assessment of endometrial thickness using TVUS, the most frequently used initial investigation, is only feasible in postmenopausal women, and a cutoff of ≥ 5 mm has a sensitivity of 96.2% and specificity of 51.5%.^7^ In Black women, the performance is poor and offers a sensitivity of only 43.7%.^8^ A positive triage result needs be followed up by histologic diagnosis, such as via hysteroscopic assessment and endometrial sampling. The low specificity of TVUS as an initial triage test, therefore, results in potentially high numbers of unnecessary invasive follow-up procedures.

Alternatives to ultrasound investigation using molecular testing have been developed. A blood-based test using cell-free DNA sequencing has reported sensitivities of 28% and 16.7% for detecting overall and stage I uterine cancers, respectively.^9^ As summarized in our recent review,^10^ we^11-13^ and others^14-22^ have previously assessed DNA-based markers in cervicovaginal samples for EC detection. DNA methylation changes in cancer appear across an entire region rather than single sites, for example, all cytosine nucleotides followed by guanine nucleotides (CpGs) in a given CpG island may be hypermethylated. This results in a substantially increased signal-to-noise ratio compared with single point mutation analysis.^23^ Sensitivities using molecular methods in cervical samples reached ≈80%,^22^ but previous studies were affected by various issues, including low sample numbers (most studies included < 40 EC cases), lack of independent validation, low sensitivities, and no standardized test format that could be applied in samples obtained via different collection strategies.

A simple test to triage women with improved, or at least equivalent, accuracy to current standards (eg, TVUS) without the need for specialist referral is urgently needed. Ideally, such a test should also be applicable across different sample types, including self-collected samples. Here, we developed and applied the Women's cancer risk IDentification – quantitative polymerase chain reaction test for Endometrial Cancer (WID-qEC) in several different settings. The test is based on quantitative, methylation-specific polymerase chain reaction (PCR) targeting one region in the gene *ZSCAN12* and two regions in *GYPC*. Our data indicate that the WID-qEC may be amenable for use in self-collected samples and could improve triage and earlier diagnosis by reducing the need for in-person consultations and invasive testing caused by low specificity of TVUS. Future prospective studies comparing TVUS and the WID-qEC side-by-side will guide clinical application of this methylation-based test.

## METHODS

### Study Population

#### Development sets.

We identified the most informative regions in an epigenome-wide screen in cervicovaginal specimens from 716 women and developed the PCR-based WID-qEC test in 40 individuals (FORECEE Pilot), 30 of whom were present in the epigenome-wide screen (total N = 726).

#### Validation sets.

**FIG 1.**
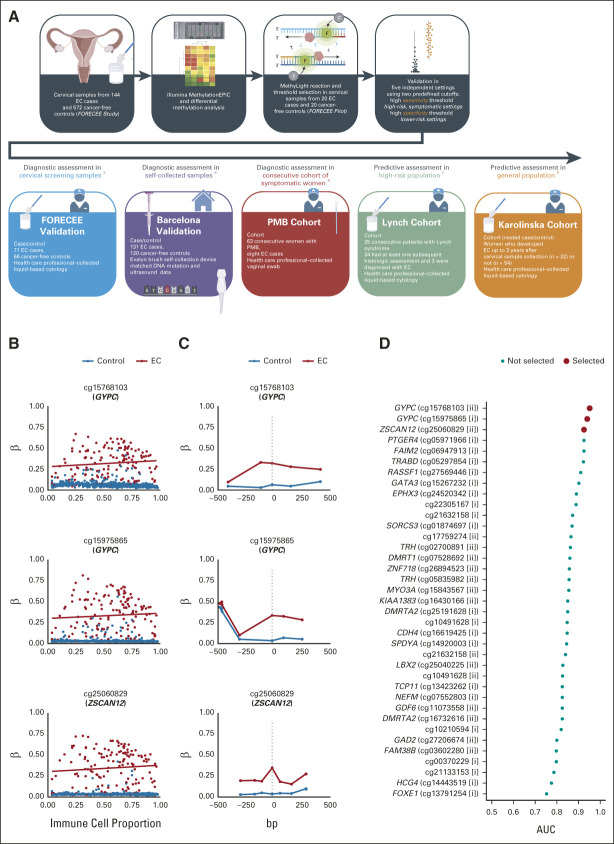
Overview of the study and selection of WID-qEC targets. (A) Schematic workflow of WID-qEC development from sample to assessment. The test was developed by epigenome-wide analysis of cervicovaginal samples from cancer cases and controls, and thresholds were fixed in a small pilot set. The test was then validated in five independent validation sets using two predefined thresholds: ^a^a high-sensitivity threshold was applied in high-risk and/or symptomatic settings (FORECEE Validation, Barcelona Validation, PMB Cohort, and Lynch Cohort), whereas ^b^a high-specificity threshold was applied in lower-risk settings (Karolinska Cohort). (B) Example plots of selected CpG beta (methylation) values in control samples and EC cases versus immune cell proportion, and (C) CpGs in their proximity (within ±500 bp). (D) AUC values of individual MethyLight reactions for discrimination of controls and cancer cases in the FORECEE Pilot set. The top three reactions, ranked by AUC, were selected for further analysis. AUC, area under the curve; bp, base pair; CpG, cytosine nucleotide followed by guanine nucleotide; EC, endometrial cancer; PMB, postmenopausal bleeding; WID-qEC, Women's cancer risk IDentification – quantitative polymerase chain reaction test for Endometrial Cancer.

We validated the test in an independent group of 562 volunteers (all > 18 years) in three diagnostic and two predictive settings (Fig 1 and Table 1): (1) the FORECEE Validation set, consisting of cervicovaginal liquid-based cytology samples from symptomatic women attending the hospital diagnosed as ECs (n = 71), benign gynecologic patients (n = 29), or healthy volunteers (n = 37), matched to cases by age; (2) the Barcelona Validation set, consisting of cervicovaginal self-samples from consecutive incident EC cases (n = 131), hospital controls with benign conditions (n = 102), and women attending hospital for nongynecologic diseases (n = 18), frequency-matched to cases by age; (3) the postmenopausal bleeding (PMB) Cohort, consisting of vaginal swabs from consecutive women presenting with postmenopausal bleeding at University College London Hospital (N = 63); (4) the Lynch Cohort, consisting of cervicovaginal liquid-based cytology samples collected from consecutive women presenting to University College London Hospital because of Lynch syndrome (N = 25); and (5) the Karolinska Cohort, a nested case/control setting using cervicovaginal liquid-based cytology samples collected between 2011 and 2015 from a Swedish cervical sample cohort–based biobank from women diagnosed with EC up to 3 years after sample collection (n = 32) or women who did not develop EC by the end of the study period (2015) on the basis of the Swedish cancer registry (n = 54).

**TABLE 1.**
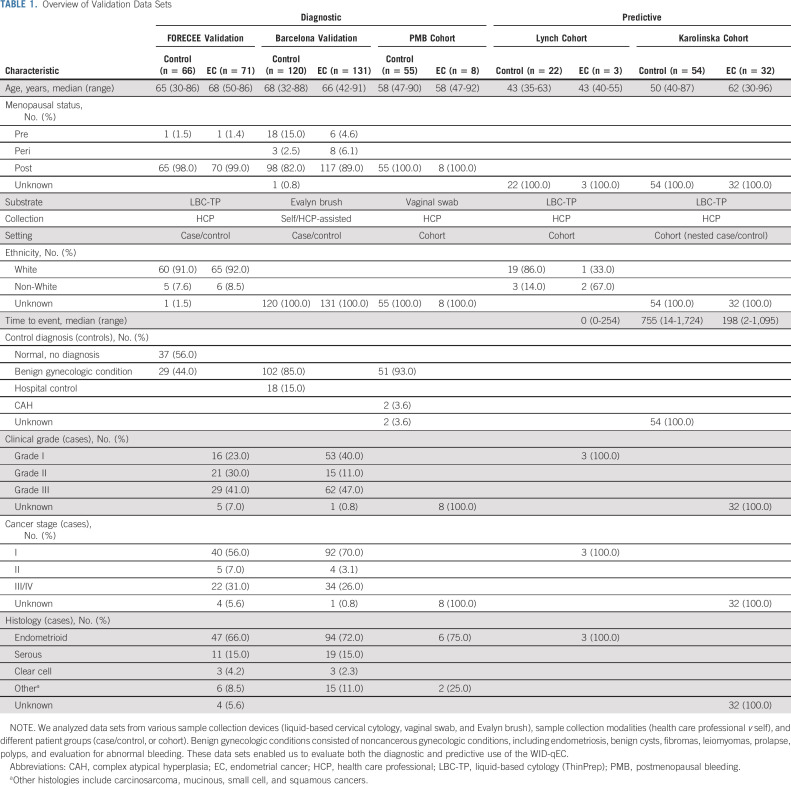
Overview of Validation Data Sets

Detailed descriptions, including inclusion criteria and sampling methodology and STARD-2015 diagrams, are provided in the Data Supplement (online only).

### Reference Test

For all EC cases, histology data following biopsy or hysterectomy were available to confirm diagnosis and deemed as the reference standard. For controls, histology was not always available. In addition to STARD diagrams, the Data Supplement details where confirmation of diagnosis using reference standard was available. Briefly, most controls in the FORECEE study did not undergo biopsy/histology, except for controls with benign gynecologic conditions. In the Barcelona Validation set, all controls with benign gynecologic conditions underwent biopsies while hospital controls (attending for other conditions) did not. In the PMB Cohort, only individuals with abnormal ultrasound underwent biopsies and histologic confirmation, while the rest without ultrasound abnormalities did not. All individuals in the Lynch Cohort, except for one participant who refused, underwent a biopsy and histologic assessment. In the Karolinska Cohort, only EC cases underwent biopsies.

### WID-qEC DNA Methylation Assay

WID-qEC test regions were discovered in an epigenome-wide approach (approximately 850,000 methylation sites). Cervicovaginal samples from 572 controls and 144 women with EC (FORECEE study) were subjected to the Illumina MethylationEPIC array following a previously established pipeline.^24^ The epigenome-wide study and details of the methylation-specific PCR-based MethyLight assay^11^ are described in the Data Supplement.

### DNA Mutation Analysis

Details of DNA mutation analysis are described in the Data Supplement. Briefly, DNA mutation analysis was performed for five genes (*PTEN*, *TP53*, *PIK3CA*, *ARID1A*, and *CTNNB1*) that led to the highest sensitivity of identifying cancers (ie, 92.9%) in The Cancer Genome Atlas data set, as previously described.^25^

### Statistical Methods

Test performance was evaluated using sensitivity, specificity, and estimates of negative and positive predictive values (NPV/PPV). Details for statistical methods are presented in the Data Supplement. Original data are available in the Data Supplement, and all analysis code is available on github and is archived on Zenodo.^26^

## RESULTS

### WID-qEC Test Development

The workflow for WID-qEC test development and assessment is shown in Figure 1. A detailed overview of the test development and threshold selection is provided in the Data Supplement. For validation of the WID-qEC in five independent sets, two fixed thresholds were defined during test development: a high-sensitivity threshold (threshold 1) was applied in symptomatic and/or high-risk settings (FORECEE Validation, Barcelona Validation, PMB Cohort, and Lynch Cohort), whereas a high-specificity threshold (threshold 2) was applied in lower-risk settings (Karolinska Cohort) where it is important to limit false-positive results. We evaluated test performance in several sample sets representing clinically relevant potential applications and report the sensitivity, specificity, and estimated PPV and NPV in each setting. Analytical covariates include age, menopausal status (pre and post), ethnicity (White *v* non-White), and cancer characteristics (stage, histology, grade, and mutation mismatch repair [MMR] status).

### WID-qEC in Cervical Smear Samples

Validation of the WID-qEC in 137 cervical smear samples (FORECEE Validation) led to a 97% sensitivity at 76% specificity and—assuming a prevalence of 9% in symptomatic women—PPV and NPV of 28% and 100%, respectively (Table 2). The results were similar, with overlapping confidence intervals, across ages, stages, grades, histologies, and menopausal status (Table 3, Data Supplement). Although sample numbers in non-White women were limited, WID-qEC performance did not seem to vary between Whites and non-Whites (Data Supplement).

**TABLE 2.**
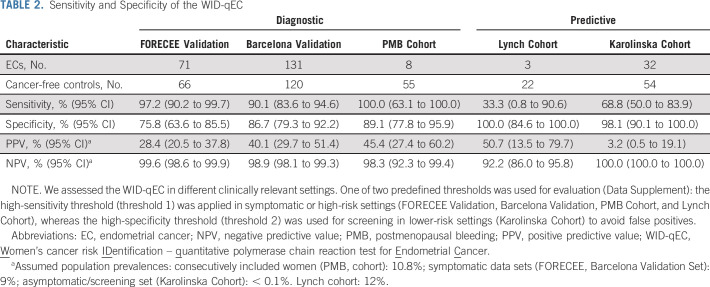
Sensitivity and Specificity of the WID-qEC

**TABLE 3.**
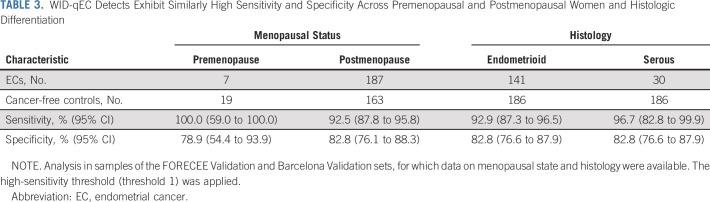
WID-qEC Detects Exhibit Similarly High Sensitivity and Specificity Across Premenopausal and Postmenopausal Women and Histologic Differentiation

### WID-qEC in Self-Collected Samples

When in-person physical access to medical facilities is restricted, a triage test ruling out malignancy would benefit from the option of sample self-collection. Among 251 (self-) Evalyn brush-collected samples (Barcelona Validation), 90% of ECs were correctly identified at 87% specificity (Table 2). The results were similar across different ages, menopausal statuses, or cancer stages, histologies, or grades (Table 3, Data Supplement). One hundred twenty-one women provided a truly self-collected sample, whereas for 130 women, health care professionals assisted with the collection using an Evalyn brush. WID-qEC performance was similar at detecting ECs in the two groups (Data Supplement).

For a subset of EC cases in this set (n = 109), DNA MMR status was available. The WID-qEC performed similarly in detection of MMR-proficient (n = 84) and MMR-deficient (n = 25) ECs (Data Supplement).

### Comparison of WID-qEC, DNAmut, and Ultrasound

Ultrasound assessment is the most common initial assessment for suspected ECs, and DNA mutations in cervicovaginal samples were recently shown to indicate ECs.^15^ In the Barcelona Validation set, ultrasound and DNA mutation data were available for subsets of women in addition to the reference standard (histology; Data Supplement), which enabled an initial comparison of the WID-qEC with different triage modalities.

**FIG 2.**
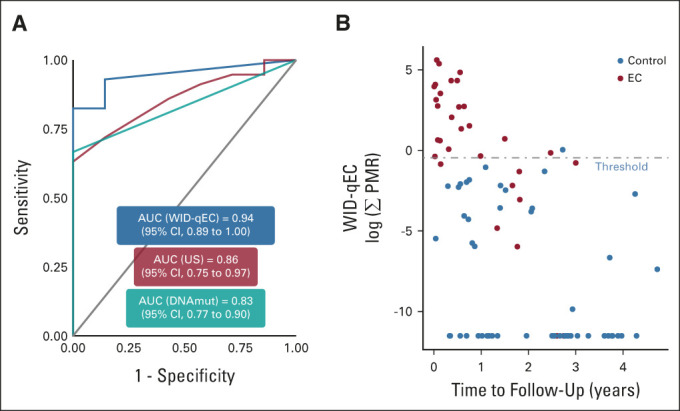
WID-qEC performance in comparison with alternative detection methods and time to diagnosis. (A) The WID-qEC exhibits a higher area under the curve than DNA mutation analysis (*P* = .002 in DeLong's test) in the Barcelona Validation set. Comparison with ultrasound offers a higher AUC point estimate and improved receiver operating characteristic curve profile (Data Supplement). Data on WID-qEC, ultrasound, and DNA mutation analysis are numerical (Σ PMR, millimeter thickness, and number of mutations, respectively). (B) The WID-qEC identifies a majority of ECs from cancer-free controls in cervical samples taken up to 3 years before diagnosis or follow-up in the Karolinska Cohort (Data Supplement). The applied threshold is the high-specificity threshold (threshold 2). AUC, area under the curve; DNAmut, DNA mutation; EC, endometrial cancer; PMR, percentage methylated reference; US, ultrasound; WID-qEC, Women's cancer risk IDentification – quantitative polymerase chain reaction test for Endometrial Cancer.

The WID-qEC offered similar sensitivity but significantly increased specificity compared with qualitative ultrasound assessment (ie, evaluation by the physician performing the ultrasound of normal or abnormal endometrial thickness; Data Supplement). AUCs for the WID-qEC score and quantitative ultrasound data (ie, endometrial thickness in millimeter) were similar (Fig 2A), but the WID-qEC exhibited higher specificity at high sensitivity than ultrasound on ROC curves (Fig 2A, Data Supplement). Moreover, compared with DNA mutation analysis (cutoff ≥ 1 mutation), the WID-qEC offered similar specificity but significantly increased sensitivity (Data Supplement) and a significantly higher AUC (Fig 2A). The same results (ie, higher specificity of WID-qEC compared with qualitative ultrasound data, and higher sensitivity compared with DNA mutation analysis) were exhibited in the analysis of stage 1 ECs only (Data Supplement). Taken together, our data indicate that the WID-qEC outperformed DNA mutation analysis and performed at least equally well as ultrasound in the Barcelona Validation set.

### WID-qEC in a Consecutive Cohort of Women With PMB

To assess the test in a real-life setting, we recruited 63 women who presented consecutively because of postmenopausal bleeding at an outpatient clinic (PMB Cohort). Health care professionals in the clinic collected a vaginal swab sample from the posterior fornix. The WID-qEC correctly identified 100% of women who were subsequently diagnosed with EC at 89% specificity (n = 8, Table 2).

### WID-qEC in a Cohort of Women With Lynch Syndrome

To assess whether the WID-qEC identifies not only women with cancer, but also women at risk of developing cancer because of genetic predisposition, we analyzed a cohort of 25 women with Lynch syndrome who consecutively attended a surveillance clinic (Lynch Cohort). The WID-qEC was negative in all women who did not show any signs of cancer at the time of sample collection. In the single patient with a positive WID-qEC test (Data Supplement), the concurrent biopsy detected an endometrioid stage 1, grade 1 EC. The sensitivity of the WID-qEC was lower in this group compared with other settings (33%, Table 2). Interestingly, two out of three cancer cases also exhibited negative histology at the time of cervical sample collection.

### WID-qEC to Predict Future Cancer Risk

Finally, we wanted to assess whether the WID-qEC detects EC in advance of current diagnosis. In the Karolinska Cohort, 69% of ECs were identified up to 3 years in advance of their diagnosis (Table 2). Sensitivity was significantly better in samples collected < 1 year before diagnosis or follow-up compared with ≥ 1 year (Fig 2B, Data Supplement). In samples collected < 1 year before last follow-up, 91% of ECs were detected with a specificity of 100%, compared with 20% detection ≥ 1 year to follow-up. These results suggest that the WID-qEC may have the potential to enable targeted, more frequent monitoring of those individuals at risk of being diagnosed with EC, although longitudinal samples in prospective studies will be required to confirm this finding.

## DISCUSSION

At the outset of this study, we aimed to develop a noninvasive EC screening and triage test. Here, we describe the WID-qEC, a simple three-marker DNA methylation–based test. We evaluate the test in clinically relevant diagnostic and predictive settings using various sample collection devices (cervical smear, vaginal swab, and self-collected samples). Applying prespecified thresholds across settings and collection modalities, our data indicate that the WID-qEC performs at least equally well, if not better, than other strategies currently in use to screen and triage women with EC, importantly ultrasound investigation.

Previous proof-of-concept studies, albeit using limited sample numbers, demonstrated the feasibility of discriminating between EC and benign conditions using genomic or epigenomic testing on Pap brush samples, tampons, or vaginal self-samples.^11-17,19-21^ Wang et al^22^ used a sensitive targeted sequencing method evaluating 18 genes of interest in a large case-control study (382 cancer cases and 714 controls): 81% of EC cases were identified in Pap brush samples. However, the specificity of this approach remains uncertain as controls were substantially younger than patients with EC (average age 34 *v* 62 years, respectively)^22^ and aging is strongly associated with the accumulation of somatic mutations.^27^ We used matched controls and observed high performance across study designs and populations. WID-qEC detection of ECs did not seem to vary by histology, grade, stage, age, ethnicity, and menopausal status in the cohorts studied here. However, future large-scale prospective studies will be required to further strengthen these data, in particular for covariates for which sample numbers in the current study were small (eg, premenopausal status, non-White ethnicities). Our data indicate that the WID-qEC could be suitable for use with a variety of collection devices. This, in combination with the high sensitivity and specificity of the test particularly in symptomatic settings, could make the WID-qEC test especially valuable in conditions where access to specialists or even any health care professional might be restricted (eg, global pandemic, nonurban settings, or in case of lengthy referral times).

The inherent limitations of case-control studies also apply to our study. We strived to mitigate bias by inclusion of several study designs (including cohorts) to ensure the robustness of the findings. We also included different sample collection methods to enhance generalizability and evaluate the potential of self-collection. Thresholds fixed during test development in the small FORECEE Pilot set detected ECs in all other sample sets and support the generalizability of our findings. A further limitation relates to the calculation of PPVs and NPVs, which were derived from the *assumed* population prevalence, meaning that changes in the assumed population prevalence could alter the estimated PPV/NPV. We note that although the PPV was sensitive to changes in estimated prevalence, the NPV remained relatively stable (Data Supplement). Nonetheless, diagnostic accuracy including PPV/NPV of the WID-qEC should ultimately be evaluated in large-scale prospective clinical studies in each target population. A limitation in the predictive evaluation using the Karolinska Cohort was that diagnoses beyond the end of 2015 were not known; hence, some controls could potentially have been diagnosed with EC after the last follow-up. Finally, we were unable to analyze 40/291 (13.7%) self-collected samples in the Barcelona Validation set because of insufficient DNA. Low yield is a common problem with self-sampling methods, but could be addressed with clear sampling guidance and optimized extraction protocols.

Two predefined diagnostic thresholds (high sensitivity or high specificity) were applied depending on the clinical context of each sample set. The appropriate threshold for clinical implementation depends on the setting: in low-risk populations, false positives should be avoided as they lead to unnecessary invasive procedures. Thus, a high-specificity approach is suitable (threshold 2). Conversely, in high-risk and/or symptomatic populations, high sensitivity is desired (threshold 1). Women who might benefit most from the WID-qEC in the near future might be (1) women presenting with abnormal bleeding or other symptoms suggestive of ECs undergoing triage for malignancies, in particular those for whom currently available tests (eg, ultrasound) are less reliable.^8^ For example, although the number of non-White women in our settings was low, the performance of the WID-qEC to detect EC was similar in White and non-White women; and (2) women at high risk of developing EC. The WID-qEC exhibited a high NPV in all settings.^10^

Pelvic ultrasound costs in the United States range from $220 US dollars (USD) to $3,200 USD, with a national average cost of $575 USD.^28^ As a relatively low-cost PCR-based test (estimated costs below $200 USD) with the potential for self-collection of samples, thus reducing the need for specialist referral, the WID-qEC offers several benefits compared with current clinical practice.

Initial clinical implementation of the WID-qEC test in circumstances where TVUS is inconclusive, not available, or declined by the patient is warranted to triage and prioritize patients with the highest cancer risk for hysteroscopy and endometrial biopsy. Given the potential benefits of earlier diagnosis of ECs for survival and reduced health care costs, future studies could also evaluate the test's potential for screening of asymptomatic women with increased risk, for instance women with obesity or Lynch syndrome, or women in the general population age > 50 years who are participating in routine cervical screening.

In conclusion, the WID-qEC could represent a patient-friendly test for the screening and triage of women with symptoms suggestive of EC or those at risk of EC. Because of its suitability for use in self-collected samples, the WID-qEC may be a suitable tool for managing women with abnormal bleeding, particularly when access to specialist care is restricted. Further research will determine the most appropriate preventive screening and early detection settings in which to deploy this test.

## Data Availability

Epigenome-wide array data are deposited in the European Genome-Phenome Archive under the accession numbers EGAS00001005033 and EGAS00001005055. Data used for this manuscript are available in the Data Supplement, and analysis source code on GitHub (https://github.com/chiaraherzog/WID-qEC-source-code).
